# Prolonged second stage of labor and risk of postpartum hemorrhage in nullipara with epidural anesthesia and vaginal delivery: A cohort study with propensity score analysis

**DOI:** 10.1002/ijgo.15816

**Published:** 2024-08-02

**Authors:** Shuang Liang, Wenguang Zheng, Ying Zhao, Baotong Su, Hongyan Cui, Yan Lv, Yanjiu Jia, Xu Chen

**Affiliations:** ^1^ Tianjin Central Hospital of Gynecology Obstetrics/Nankai University Affiliated Maternity Hospital Tianjin China; ^2^ Tianjin Key Laboratory of Human Development and Reproductive Regulation Tianjin China; ^3^ School of Computer Science and Engineering Tianjin University of Technology Tianjin China; ^4^ Tianjin Key Laboratory of Intelligence Computing and Novel Software Technology Tianjin University of Technology Tianjin China; ^5^ School of Medicine Nankai University Tianjin China

**Keywords:** labor, overlap weighting, postpartum hemorrhage, prolonged second stage, second stage of labor

## Abstract

**Objective:**

To conduct an analysis using propensity score methods, exploring the association between a prolonged second stage (>3 h) and the risk of postpartum hemorrhage (PPH) in a diverse population.

**Methods:**

We conducted a prospective cohort study involving nullipara with epidural anesthesia and vaginal delivery, aged ≥18 years, presenting cephalically, and with a gestational age (GA) of ≥24 weeks at a tertiary maternity hospital in China (chictr.org.cn identifier: ChiCTR2200063094). Women undergoing emergency cesarean section in labor were excluded. The primary outcome was PPH, with secondary outcomes including severe postpartum hemorrhage and blood transfusion. We employed propensity score overlap weighting to analyze the association between prolonged second stage labor and PPH.

**Results:**

The study included 3643 nullipara with epidural anesthesia, comprising 77 with a second stage of labor >3 h and 3566 with a second stage ≤3 h. Utilizing propensity score overlap weighting, there were no significant differences observed between the two groups regarding the risk of PPH (29.87% in >3 h group vs 17.64% in ≤3 h group; weighted odds ratio 1.01; 95% CI: 0.51–2.02). Subgroup interaction tests for PPH were not significant for assisted vaginal delivery, induction of labor, macrosomia, third‐/fourth‐degree perineal laceration, GA >41 weeks, twin pregnancies, episiotomy and GA >37 weeks. Sensitivity analysis did not reveal significant differences.

**Conclusion:**

This study did not find evidence supporting an increased risk of PPH associated with a second stage of labor lasting >3 h in our population, providing additional evidence for clinical practice.

## Synopsis

No significant relationship between the prolonged second stage of labor and the risk of PPH was found even after employing overlap weighting in our population.

**Abbreviations**
CS: cesarean section
GA: gestational age
PPH: postpartum hemorrhage
sPPH: severe postpartum hemorrhage

## 1 INTRODUCTION

At the turn of this century, the longstanding duration of the second stage of labor, which remained at 2 h for two centuries, underwent revision. However, several unresolved issues persist in this domain.^1^, ^2^ With continuous advancements in prenatal care, including fetal monitoring and epidural delivery, there is a need to redefine the duration of the second stage of labor to reduce excessive interventions during delivery. Building upon the retrospective observational study by Zhang et al., which focused on a large sample of low‐risk individuals with normal fetal outcomes, current recommendations from the American College of Obstetricians and Gynecologists (ACOG) now allow 4 h for the second stage of labor in nullipara and 3 h in multiparous women with epidural anesthesia.^3^ However, it remains uncertain whether a prolonged duration of the second stage of labor (beyond 3 h with epidural anesthesia or 2 h without epidural anesthesia) contributes to adverse maternal outcomes, such as postpartum hemorrhage (PPH), which is the leading cause of maternal mortality and morbidity worldwide, especially in the susceptible individuals with high‐risk factors.^4^

Numerous clinical retrospective studies present conflicting evidence regarding the effect of a prolonged second stage on PPH.^5^, ^6^, ^7^, ^8^ A recent meta‐analysis of 13 retrospective studies, involving 337 845 parturients, suggested an association between a prolonged second stage (nullipara >3 h/nullipara: with epidural anesthesia >3 h, no epidural anesthesia >2 h/nullipara: 2–3 h/multipara: with epidural anesthesia >2 h, no with epidural anesthesia >1 h) and PPH but lacked effective correction for confounding factors.^9^ In contrast, Gimovsky et al. conducted a randomized controlled trial with a small sample of 78 singleton, nulliparous individuals. Participants were randomly assigned to extended labor for at least an additional hour for expedited delivery or cesarean/operative vaginal delivery when the second stage of labor was deemed “prolonged” (beyond 3 h with epidural anesthesia or 2 h without epidural anesthesia).^10^ Results indicated that a prolonged second stage decreased the incidence of cesarean section by 55%, without an increase in maternal or neonatal morbidity, including PPH. In the absence of randomized controlled studies with large samples, well‐designed prospective cohort studies with rigorous control of confounders may provide valuable evidence for clinical practice, and propensity scores, which summarize differences in patient characteristics to make groups comparable, enable control of the covariates.

To assess the association between a second stage lasting longer than 3 h and PPH, we conducted a prospective cohort study involving 3643 nullipara admitted for vaginal delivery with epidural. Utilizing propensity scores and overlap weighting, we aimed to render maternal baseline information comparable. Furthermore, to ensure the robustness of our findings, we performed subgroup analyses on eight potential variables related to PPH and conducted sensitivity analyses.

## 2 MATERIALS AND METHODS

### 2.1 Study participants

This prospective cohort study included pregnant women who were admitted to the hospital for delivery from October 1, 2022 to November 1, 2023 at Tianjin Central Hospital of Obstetrics and Gynecology (chictr.org.cn identifier: ChiCTR2200063094). Pertinent information regarding subject demographic characteristics, past medical and pregnancy history, current pregnancy history was collected by a physician and entered into an electronic case report form. Participants' weight was measured upon admission. Data on outcomes were collected up to 24 h postpartum.

Inclusion criteria comprised nullipara, maternal age of at least 18 years, cephalic presentation, and gestational age ≥24 weeks. Exclusion criteria included emergency cesarean section (CS) in labor and the absence of epidural anesthesia for pain analgesia.

### 2.2 Exposure

The exposure variable was the duration of the second stage of labor, defined as the period between full cervical dilatation and the birth of the baby.^11^ Factors likely associated with PPH were identified as covariates. These included all risk factors in the California Maternal Quality Care Collaborative (CMQCC), and additional pregnancy complications such as low‐lying placenta, twin pregnancies, assisted vaginal delivery, induction of labor, chorioamnionitis, macrosomia, retained placenta tissue, gestational age (GA) <37 weeks, GA >41 weeks, large uterine fibroids, platelets 50–100 000, maternal obesity (body mass index [BMI, calculated as weight in kilograms divided by the square of height in meters] >30), advanced maternal age (≥40 years at delivery), prior cesarean(s) or uterine surgery, polyhydramnios, hematocrit <30%, third/fourth‐degree perineal laceration^12^, ^13^, ^14^, ^15^, ^16^, ^17^ (Table S1).

### 2.3 Outcomes

The primary outcome was defined as PPH, characterized by blood loss of 500 mL or more from the genital tract within 24 h of a vaginal birth, following the 1989 WHO working group definition.^11^, ^18^ Secondary outcomes included severe postpartum hemorrhage (sPPH) and blood transfusion, with sPPH defined as blood loss of 1000 mL or more within 24 h of vaginal birth.^11^ To measure blood loss, a blood bag was used to weigh and record the amount, with a resident physician present at delivery. After delivery, a weighed cotton pad was placed under the mother's perineum. At 24 h post‐delivery, all used pads were collected, and the bleeding pads were weighed in grams. The total weight for each case was divided by the specific gravity of blood (1.05) to obtain the volume. Blood transfusion was defined as the transfusion of at least 1 unit of packed red blood cells (RBCs) before discharge.

### 2.4 Statistical analysis

Data were analyzed from November 1 to December 2, 2023. Categorical variables were compared using the Chi squared or Fisher exact tests, while continuous variables were compared using the two‐tailed student *t*‐test or Wilcoxon rank‐sum test. The second stage of labor >3 h was considered a prolonged second stage (>3 h group), and ≤3 h was considered a normal second stage (≤3 h group).^2^

Primary and secondary outcomes were compared between the >3 h group and ≤3 h groups using univariate analysis. Additionally, the duration of the second stage of labor was modeled with restricted cubic splines to account for nonlinear relationships with the outcomes. A *P* value of less than 0.05 was considered statistically significant.

To address potential selection bias, propensity scores were developed to balance the probability of experiencing the exposure (second stage of labor >3 h) in the two groups. Based on propensity scores, two adjustment approaches, overlap weighting, and propensity score matching (PSM) 1 to 1, 2, 3, and 4 matching (with a caliper width of 0.1), were performed to examine the association between exposure and PPH, considering individual covariates. The covariates included baseline maternal sociodemographic and clinical factors expected to be associated with PPH.

Overlap weighting was selected as the optimal adjustment method based on standardized mean differences, ensuring a balance of less than 10% for all covariates (Figure S1). Subsequently, univariate logistic regression was employed to estimate odds ratios and 95% confidence intervals for the association between exposure and PPH.

Exploratory post hoc subgroup analyses were conducted across eight variables: assisted vaginal delivery, induction of labor, macrosomia, third‐/fourth‐degree perineal laceration, GA >41 weeks, twin pregnancies, episiotomy and GA >37 weeks. A post hoc sensitivity analysis was also performed. First, factors potentially on the causal pathway between prolonged second stage and PPH—assisted vaginal delivery, episiotomy, and third‐/fourth‐degree perineal laceration were excluded from the covariates. Second, the 926 excluded women who underwent emergency CS in labor were added to the analysis population.

All statistical analyses were conducted with R statistical software, version 4.2.2 (R Project for Statistical Computing).

### 2.5 Ethics statement

The study received approval from the Ethics Committee of Tianjin Central Hospital of Obstetrics and Gynecology (approval ID: 2022KY063, granted on August 2, 2022). Informed consent was obtained from all participating women during prenatal care consultations before the commencement of the second stage.

Our study adhered to the Strengthening the Reporting of Observational Studies in Epidemiology (STROBE) reporting guideline for cohort studies. Additionally, the trial was registered with the Chinese Clinical Trial Registry under the identifier ChiCTR2200063094, with the registration date of August 30, 2022. The initial participant enrollment for the trial commenced on October 1, 2022. Further details can be accessed at http://www.chictr.org.cn/enindex.aspx.

## 3 RESULTS

**FIGURE 1:**
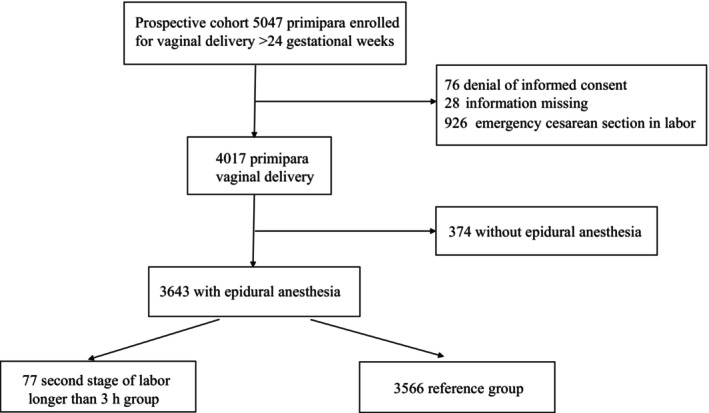
Description of the study cohort.

A total of 5047 patients were initially assessed for eligibility, and 1404 were excluded (76 due to the denial of informed consent, 28 with missing information, 926 undergoing emergency CS in labor, of which two had a second stage of labor >3 h, and 374 without epidural anesthesia). Consequently, the final analysis included 3643 individuals (72.18% of the total), comprising 77 cases in the >3 h group and 3566 cases in the ≤3 h group (Figure 1; Table 1). Among the 77 cases, seven (9.09%) had a second stage of labor >4 h, and 70 (90.91%) had a second stage of labor between 3 and 4 h. The overall prevalence of PPH was 17.90% (652/3643).

**TABLE 1:** Baseline characteristics of the study cohort.

Characteristics	Second stage >3 h No. (%) (*n* = 77)	Second stage ≤3 h No. (%) (*n* = 3566)	*P* value	Absolute standardized mean difference
Maternal characteristics				
Maternal age, mean (SD), year	29.55 (3.41)	29.34 (3.69)	0.620	
Age ≥40	0	19 (0.53%)	0.521	0.000
BMI	26.73 (3.56)	27.27 (6.36)	0.549	
Obesity (BMI≥30)	13 (16.88%)	710 (19.91%)	0.509	0.006
Pre‐existing diabetes/chronic hypertension	0	26 (0.73%)	0.452	0.000
Prior cesarean(s) or uterine surgery	0	14 (0.39%)	0.582	0.000
Uterus malformation	0	9 (0.25%)	0.659	0.000
Pregnancy				
Thrombocytopenia	0	6 (0.17%)	0.719	0.000
Twin pregnancies	1 (1.30%)	15 (0.42%)	0.249	0.011
IVF‐ET	5 (6.49%)	169 (4.74%)	0.476	0.016
Anemia (Hct <0.3)	0	67 (1.88%)	0.225	0.000
PE	1 (1.30%)	45 (1.26%)	0.977	0.005
GDM	13 (16.88%)	638 (17.89%)	0.771	0.001
Stillbirth	1 (1.30%)	26 (0.73%)	0.564	0.005
Polyhydramnios	1 (1.30%)	53 (1.49%)	0.893	0.000
Large fibroid	1 (1.30%)	59 (1.65%)	0.459	0.005
Placenta previa/low lying placenta	1 (1.30%)	18 (0.50%)	0.532	0.000
Labor				
PTB	2 (2.60%)	247 (6.93%)	0.136	0.002
GA >41	12 (15.58%)	241 (6.76%)	0.003	0.000
Induction of labor	40 (51.95%)	1281 (35.92%)	0.004	0.008
Chorioamnionitis	2 (2.60%)	109 (3.06%)	0.816	0.006
Birth				
Retained placenta	4 (5.19%)	170 (4.77%)	0.863	0.01
Macrosomia (>4000 g)	5 (6.49%)	114 (3.20%)	0.108	0.005
Assisted vaginal delivery	18 (23.38%)	181 (5.08%)	0.000	0.004
Episiotomy	54 (70.13%)	1273 (35.70%)	0.000	0.003
Third‐/fourth‐degree perineal laceration	5 (6.49%)	116 (3.25%)	0.117	0.000

Abbreviations: GA, gestational age; GDM, gestational diabetes mellitus; IVF‐ET, in vitro fertilization and embryo transfer; PE, pre‐eclampsia; PTB, preterm birth.

Baseline characteristics of the two groups are presented in Table 1. Factors such as GA >41 weeks, assisted vaginal delivery, induction of labor, and episiotomy were more prevalent in the >3 h group (*P* < 0.05). Other factors did not exhibit significant differences between the two groups.

The association between the duration of the second stage and PPH was examined using restricted spline curves (RSC), treating duration as a continuous variable. The results indicated a nonlinear increasing trend with the duration of the second stage for PPH (*P* = 0.000). Similar trends were observed for the risk of severe postpartum hemorrhage (sPPH) (*P* = 0.018). However, no significant difference was found in the risk of blood transfusion (*P* = 0.260) (Figure S2).

In binary logistic regression analysis using the duration of the second stage as a categorical variable, it was observed that a second stage lasting >3 h was associated with an increased risk of PPH (OR 2.00; 95% CI: 1.22–2.38). However, this association was not observed for sPPH (OR 0.61; 95% CI: 0.08–4.46) or blood transfusion (OR 0.86; 95% CI: 0.12–6.27) (Table 2).

**TABLE 2:** The association between second stage >3 h and the outcome.

Outcome	Crude (unweighted)				After overlap weighting			
	Second stage >3 h *N* (%) (*n* = 77)	Second stage ≤3 h *N* (%) (*n* = 3566)	OR (95% CI)	*P* value	Second stage >3 h (%)	Second stage ≤3 h (%)	OR (95% CI)	*P* value
PPH	23 (29.87%)	629 (17.64%)	2.00 (1.22–3.28)	0.006	29.87	29.87	1.01 (0.51–2.02)	0.971
sPPH	1 (1.30%)	74 (2.08%)	0.61 (0.08–4.46)	0.628	1.30	3.90	0.33 (0.03–3.26)	0.343
Blood transfusion	1 (1.30%)	53 (1.49%)	0.86 (0.12–6.27)	0.878	1.30	2.60	0.46 (0.04–5.10)	0.534

Abbreviations: CI, confidence interval; OR, odds ratio; PPH, postpartum hemorrhage; sPPH, severe postpartum hemorrhage.

Following overlap weighting, the univariate logistic regression analysis revealed no significant differences between the >3 h group and ≤3 h group in terms of the risk of PPH (29.87% in the >3 h group vs 17.64% in the ≤3 h group; weighted OR 1.01; 95% CI: 0.51–2.02). Similar results were observed for the risk of sPPH (weighted OR 0.33; 95% CI: 0.03–3.26) and blood transfusion (weighted OR 0.46; 95% CI: 0.04–5.10) (Table 2).

**FIGURE 2:**
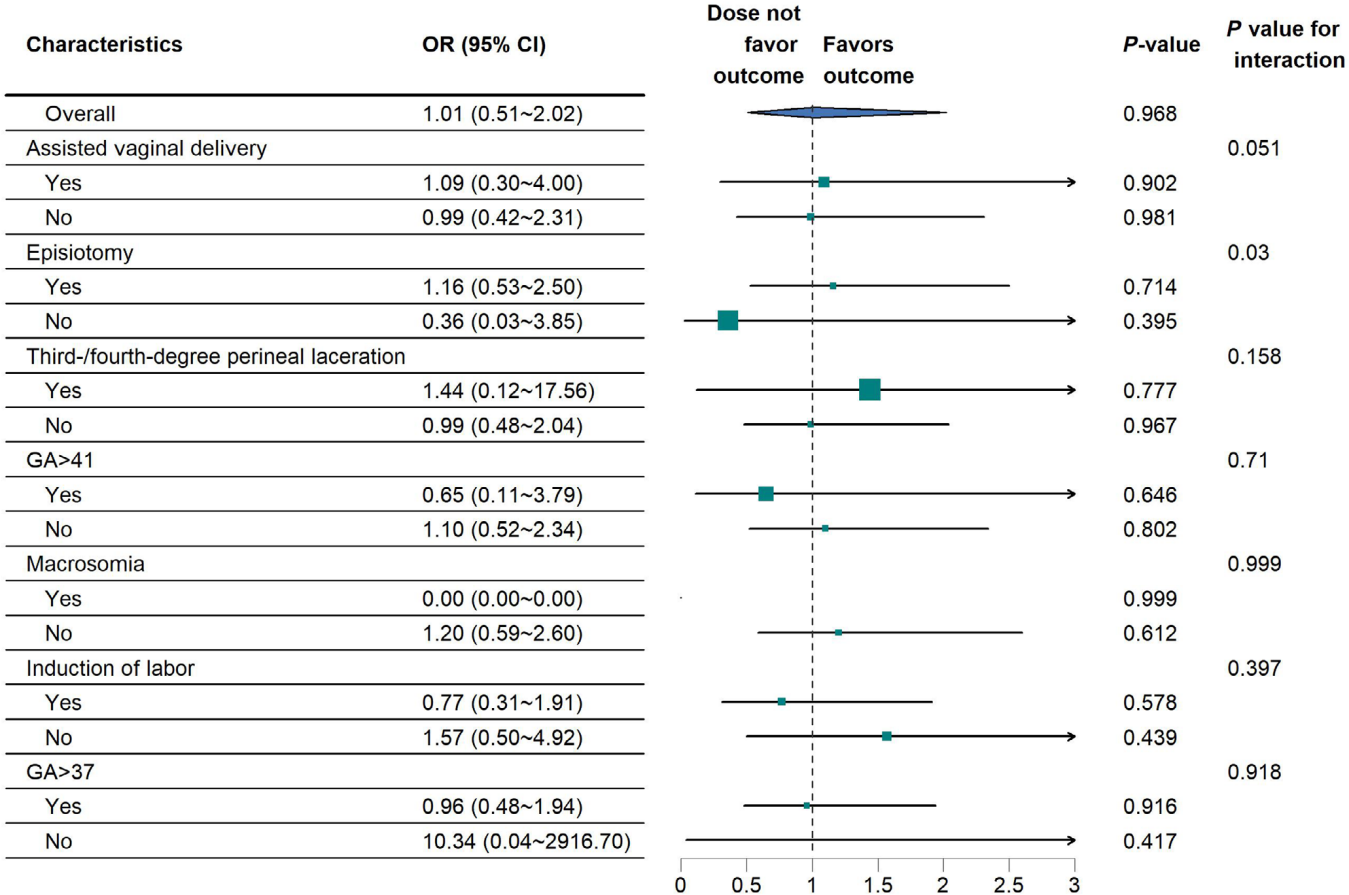
Association between second stage >3 h and postpartum hemorrhage in the prespecified subgroups after overlap weighting. GA, gestational age.

Subgroup analysis demonstrated no significant interaction for PPH between a second stage of labor >3 h and other factors, including assisted vaginal delivery, induction of labor, macrosomia, third‐/fourth‐degree perineal laceration, GA >41 weeks, twin pregnancies, episiotomy and GA >37 weeks (Figure 2). The sensitivity analyses yielded consistent results regarding the lack of association of a second stage of labor >3 h with PPH after excluding assisted vaginal delivery, episiotomy and third‐/fourth‐degree perineal laceration from the overlap weighting (OR 1.67; CI: 0.80–3.48) (Table 3) and after adding the 926 excluded women who underwent emergency CS in labor (OR 1.00; CI: 0.51–1.99) (Table 4).

**TABLE 3:** The association between second stage >3 h and the outcome after excluding assisted vaginal delivery, episiotomy and third‐/fourth‐degree perineal laceration from the overlap weighting.

Outcome	Crude (unweighted)				After overlap weighting			
	Second stage >3 h, *N* (%) (*n* = 77)	Second stage ≤3 h, *N* (%) (*n* = 3566)	OR (95% CI)	*P* value	Second stage >3 h (%)	Second stage ≤3 h (%)	OR (95% CI)	*P* value
PPH	23 (29.87%)	629 (17.64%)	2.00 (1.22–3.28)	0.006	29.87	20.78	1.67 (0.80–3.48)	0.177
sPPH	1 (1.30%)	74 (2.08%)	0.61 (0.08–4.46)	0.628	1.30	2.60	0.55 (0.05–6.44)	0.633
Blood transfusion	1 (1.30%)	53 (1.49%)	0.86 (0.12–6.27)	0.878	1.30	1.30	0.73 (0.05–9.91)	0.816

Abbreviations: CI, confidence interval; OR, odds ratio; PPH, postpartum hemorrhage; sPPH, severe postpartum hemorrhage.

**TABLE 4:** The association between second stage >3 h and the outcome after adding emergency cesarean section during labor.

Outcome	Crude (unweighted)				After overlap weighting			
	Second stage >3 h, *N* (%) (*n* = 79)	Second stage ≤3 h *N* (%) (*n* = 4490)	OR (95% CI)	*P* value	Second stage >3 h (%)	Second stage ≤3 h (%)	OR (95% CI)	*P* value
PPH	23 (29.11%)	633 (14.10%)	2.50 (1.53–4.09)	0.000	29.11	29.11	1.00 (0.51–1.99)	0.992
sPPH	1 (1.27%)	74 (1.65%)	0.75 (0.10–5.50)	0.781	1.27	3.80	0.34 (0.03–3.32)	0.350
Blood transfusion	1 (1.27%)	55 (1.22%)	1.03 (0.14–7.56)	0.974	1.27	2.53	0.47 (0.04–5.20)	0.539

Abbreviations: CI, confidence interval; OR, odds ratio; PPH, postpartum hemorrhage; sPPH, severe postpartum hemorrhage.

## 4 DISCUSSION

In this single‐center prospective cohort study, we did not find evidence supporting an increased risk of PPH associated with a second stage of labor lasting >3 h in nullipara with epidural anesthesia and vaginal delivery. This conclusion was robustly supported by subgroup interaction tests, including various factors such as assisted vaginal delivery, induction of labor, macrosomia, third‐/fourth‐degree perineal laceration, GA >41 weeks, twin pregnancies, episiotomy and GA >37 weeks. Sensitivity analyses excluding assisted vaginal delivery, episiotomy, and third‐/fourth‐degree perineal laceration from the overlap weighting and adding the 926 excluded women who underwent emergency CS in labor consistently affirmed this finding.

In our study, the prevalence of PPH (defined as bleeding loss >500 mL) was 17.90%, similar to previous studies^8^, ^19^ and higher than the known prevalence of 3%–6%,^20^, ^21^ possibly due to the differences in definition of PPH^18^, ^22^, ^23^ and the measurements of blood loss.^24^ While some studies have reported associations between a prolonged second stage and PPH, the issue remains controversial.^7^, ^25^, ^26^, ^27^, ^28^ Miller et al. conducted a case–control study, reporting an association between a second stage >3 h and PPH.^29^ Based on a population‐based cohort study, Emelie et al. reported that an increased length of the second stage (increased with each passing hour of second stage) was associated with an increased risk of PPH.^8^ Young et al., in a population‐based cohort study, found that a prolonged second stage (nullipara >3 h or multipara >2 h) was associated with an increased risk of PPH (adjusted odds ratio 1.27; 95% CI: 1.25–1.30).^5^ In our study, we observed an association between the duration of the second stage of labor and PPH, whether treated as a categorical or continuous variable before overlap weighting. However, after overlap weighting, no relationship between the duration of the second stage of labor and PPH was found, aligning with the findings of a randomized controlled trial (RCT).^10^

To address potential confounding factors in the association between the duration of the second stage of labor and postpartum hemorrhage (PPH), our study employed several strategies. First, instead of using conventional multivariable logistic regression, we utilized the propensity score overlap weighting method to effectively control for potential confounders.^30^ For covariates, we included nearly all clinical factors associated with PPH based on the CMQCC Quality Improvement Toolkit 3.0.^12^, ^13^, ^14^ Second, the robustness of our results was confirmed through multiple subgroup and sensitivity analyses, demonstrating the reliability and consistency of our findings. Third, the study design as a prospective cohort study minimized bias. Additionally, in contrast to a randomized controlled trial (RCT), our study encompassed diverse populations, enhancing its effectiveness.

This study had several limitations. First, the sample size was limited, preventing a detailed subgroup analysis of exposure factors (3–4 or >4 h in the second stage of labor) to thoroughly examine the relationship between duration and PPH. Second, the single‐center design hampers external validity, necessitating verification through multicenter studies to generalize the conclusions. Third, the exclusion of women who delivered by emergency CS in labor limited our ability to assess the impact of the second stage on PPH. While this exclusion eliminates the effect of cesarean delivery on PPH, as cesarean is a risk factor, it introduces a potential limitation.

## 5 CONCLUSIONS

In this cohort study, we did not find evidence supporting an increased risk of PPH associated with a second stage of labor lasting >3 h in nullipara with epidural anesthesia and vaginal delivery. Our findings contribute additional evidence for ACOG's management of the second stage of labor. However, further exploration through larger and better‐designed trials is needed to understand the relationship between PPH and the prolongation of the second stage of labor more comprehensively.

## AUTHOR CONTRIBUTIONS

**Shuang Liang and Xu Chen**: Conceptualized the study. **Yan Lv, Hongyan Cui, Yanjiu Jia, Baotong Su and Wenguang Zheng**: Involved in data collection and analysis. **Shuang Liang, Baotong Su, and Wenguang Zheng**: Contributed to the analysis and interpretation of results. **Shuang Liang**: Drafted the manuscript. **Xu Chen**: Revised and edited the manuscript. All authors approved the final version, acknowledging accountability for all aspects of the work, ensuring integrity and accuracy.

## FUNDING INFORMATION

This study was supported by the National Key Research and Development Program of China (2021YFC2701500), Tianjin Key Medical Discipline (Specialty) Construction Project (TJYXZDXK‐043A), Tianjin Municipal Science and Technology Project (21JCZDJC00080), The Open Fund of Tianjin Central Hospital of Gynecology Obstetrics/Tianjin Key Laboratory of human development and reproductive regulation (2022XHY01), National Clinical Key Specialty and Key Disease Queue Project (GJZDZKZBDL2022‐03), Tianjin Municipal Education Commission Research Project (2023YXYZ07).

## CONFLICT OF INTEREST STATEMENT

The authors have no competing interests to declare.

## Supporting information


Data S1.

## Data Availability

Research data are not shared.

## References

[1] Friedman EA . Primigravid labor; a graphicostatistical analysis. Obstet Gynecol. 1955;6(6):567‐589.13272981

[2] ACOG Practice Bulletin No. 154 . Operative vaginal delivery. Obstet Gynecol. 2015;126(5):e56‐e65. 26488523 10.1097/AOG.0000000000001147

[3] Zhang J , Landy HJ , Ware Branch D , et al. Contemporary patterns of spontaneous labor with normal neonatal outcomes. Obstet Gynecol. 2010;116(6):1281‐1287. 21099592 10.1097/AOG.0b013e3181fdef6e PMC3660040

[4] Bienstock JL , Eke AC , Hueppchen NA . Postpartum hemorrhage. N Engl J Med. 2021;384(17):1635‐1645. 33913640 10.1056/NEJMra1513247 PMC10181876

[5] Young C , Bhattacharya S , Woolner A , et al. Maternal and perinatal outcomes of prolonged second stage of labour: a historical cohort study of over 51,000 women. BMC Pregnancy Childbirth. 2023;23(1):467. 37349683 10.1186/s12884-023-05733-z PMC10288707

[6] Sheiner E , Sarid L , Levy A , Seidman DS , Hallak M . Obstetric risk factors and outcome of pregnancies complicated with early postpartum hemorrhage: a population‐based study. J Matern Fetal Neonatal Med. 2005;18(3):149‐154. 16272036 10.1080/14767050500170088

[7] Hyredin T , Urgie T , Sium AF . Prolonged second stage of labor: predictors of adverse maternal and perinatal outcomes in a sub‐Saharan setting. Int J Gynaecol Obstet. 2023;163(3):997‐1004. 37417324 10.1002/ijgo.14982

[8] Looft E , Simic M , Ahlberg M , Snowden JM , Cheng YW , Stephansson O . Duration of second stage of labour at term and pushing time: risk factors for postpartum haemorrhage. Paediatr Perinat Epidemiol. 2017;31(2):126‐133. 28195653 10.1111/ppe.12344

[9] Pergialiotis V , Bellos I , Antsaklis A , Papapanagiotou A , Loutradis D , Daskalakis G . Maternal and neonatal outcomes following a prolonged second stage of labor: a meta‐analysis of observational studies. Eur J Obstet Gynecol Reprod Biol. 2020;252:62‐69. 32570187 10.1016/j.ejogrb.2020.06.018

[10] Gimovsky AC , Berghella V . Randomized controlled trial of prolonged second stage: extending the time limit vs usual guidelines. Am J Obstet Gynecol. 2016;214(3):e361‐e366. 10.1016/j.ajog.2015.12.042 26928148

[11] World Health Organization . WHO Recommendations: Intrapartum Care for a Positive Childbirth Experience. World Health Organization; 2018. 30070803

[12] Dilla AJ , Waters JH , Yazer MH . Clinical validation of risk stratification criteria for peripartum hemorrhage. Obstet Gynecol. 2013;122(1):120‐126. 23743452 10.1097/AOG.0b013e3182941c78

[13] Ruppel H , Liu VX , Gupta NR , Soltesz L , Escobar GJ . Validation of postpartum hemorrhage admission risk factor stratification in a large obstetrics population. Am J Perinatol. 2021;38(11):1192‐1200. 32455467 10.1055/s-0040-1712166 PMC7688483

[14] Ladfors LV , Butwick A , Stephansson O . A validation of the California Maternal Quality Care Collaborative obstetric hemorrhage risk assessment tool in a Swedish population. Am J Obstet Gynecol MFM. 2023;6:101240. 38056628 10.1016/j.ajogmf.2023.101240

[15] Taylor K , Noel E , Chapple AG , Buzhardt S , Sutton E . Risk factors for postpartum hemorrhage in a tertiary hospital in South‐Central Louisiana. J Matern Fetal Neonatal Med. 2022;35(25):7353‐7359. 34304671 10.1080/14767058.2021.1948528

[16] Al‐Khatib A , Sagot P , Cottenet J , Aroun M , Quantin C , Desplanches T . Major postpartum haemorrhage after frozen embryo transfer: a population‐based study. BJOG. 2023;131:300‐308. 37550089 10.1111/1471-0528.17625

[17] Braund S , Deneux‐Tharaux C , Sentilhes L , Seco A , Rozenberg P , Goffinet F . Induction of labor and risk of postpartum hemorrhage in women with vaginal delivery: a propensity score analysis. Int J Gynaecol Obstet. 2023;164:732‐740. 37568268 10.1002/ijgo.15043

[18] Vogel JP , Williams M , Gallos I , Althabe F , Oladapo OT . WHO recommendations on uterotonics for postpartum haemorrhage prevention: what works, and which one? BMJ Glob Health. 2019;4(2):e001466. 10.1136/bmjgh-2019-001466 PMC6509591 31139461

[19] Medical Birth Registry of Norway. https://www.fhi.no/en/hn/health‐registries/medical‐birth‐registry‐of‐norway/

[20] Reale SC , Easter SR , Xu X , Bateman BT , Farber MK . Trends in postpartum hemorrhage in the United States from 2010 to 2014. Anesth Analg. 2020;130(5):e119‐e122. 31567319 10.1213/ANE.0000000000004424

[21] Mehrabadi A , Hutcheon JA , Lee L , Kramer MS , Liston RM , Joseph KS . Epidemiological investigation of a temporal increase in atonic postpartum haemorrhage: a population‐based retrospective cohort study. BJOG. 2013;120(7):853‐862. 23464351 10.1111/1471-0528.12149 PMC3717179

[22] Menard MK , Main EK , Currigan SM . Executive summary of the reVITALize initiative: standardizing obstetric data definitions. Obstet Gynecol. 2014;124(1):150‐153. 24901267 10.1097/AOG.0000000000000322

[23] Green‐top Guideline No. 52 . Prevention and management of postpartum haemorrhage. BJOG. 2017;124(5):e106‐e149. 27981719 10.1111/1471-0528.14178

[24] Ruiz MT , Azevedo NF , Resende CV , et al. Quantification of blood loss for the diagnosis of postpartum hemorrhage: a systematic review and meta‐analysis. Rev Bras Enferm. 2023;76(6):e20230070. 38055493 10.1590/0034-7167-2023-0070 PMC10695064

[25] Nelson DB , McIntire DD , Leveno KJ . Second‐stage labor: consensus versus science. Am J Obstet Gynecol. 2020;222(2):144‐149. 31473231 10.1016/j.ajog.2019.08.044

[26] Niemczyk NA , Ren D , Stapleton SR . Associations between prolonged second stage of labor and maternal and neonatal outcomes in freestanding birth centers: a retrospective analysis. BMC Pregnancy Childbirth. 2022;22(1):99. 35120470 10.1186/s12884-022-04421-8 PMC8815242

[27] Altman MR , Lydon‐Rochelle MT . Prolonged second stage of labor and risk of adverse maternal and perinatal outcomes: a systematic review. Birth. 2006;33(4):315‐322. 17150071 10.1111/j.1523-536X.2006.00129.x

[28] Cheng YW , Hopkins LM , Caughey AB . How long is too long: does a prolonged second stage of labor in nulliparous women affect maternal and neonatal outcomes? Am J Obstet Gynecol. 2004;191(3):933‐938. 15467567 10.1016/j.ajog.2004.05.044

[29] Miller CM , Cohn S , Akdagli S , Carvalho B , Blumenfeld YJ , Butwick AJ . Postpartum hemorrhage following vaginal delivery: risk factors and maternal outcomes. J Perinatol. 2017;37(3):243‐248. 27977018 10.1038/jp.2016.225 PMC5334143

[30] Thomas LE , Li F , Pencina MJ . Overlap weighting: a propensity score method that mimics attributes of a randomized clinical trial. JAMA. 2020;323(23):2417‐2418. 32369102 10.1001/jama.2020.7819

