# Supplementary material for: Prolonged second stage of labor and risk of postpartum hemorrhage in nullipara with epidural anesthesia and vaginal delivery: A cohort study with propensity score analysis
Source: Int J Gynaecol Obstet. 2024 Aug 2;168(1):141–8. doi: 10.1002/ijgo.15816 (PMC11649855; doi:10.1002/ijgo.15816)
Supplement: Supplementary file 1 — Data S1. [file IJGO-168-141-s001.docx]

**Supplementary Online Content**

**Table S1.** Definitions of variables

**Figure S1.** Methods compared.

**Figure S2.** The associations of second stage of labor with the outcome by restricted cubic spline regressions.

This supplementary material has been provided by the authors to give readers additional information about their work.

**Table S1. Definitions of variables.**

| **Variables** | **Definition** |
| --- | --- |
| PTB | Gestational week<37 |
| Thrombocytopenia | Platelet count< 100*109/L. |
| PE | Preeclampsia is characterized by new-onset hypertension which usually occurs after 20 weeks’ gestation, and evidence of end-organ dysfunction. |
| GDM | GDM is diagnosed if any of the 75 g OGTT plasma glucose values during 24–28 gestational weeks meets or exceeds the following cutoff values: 5.1 mmol/L at fasting; 10.0 mmol/L at 1 h; and 8.5 mmol/L at 2 h |
| Polyhydramnios | Amniotic fluid index (AFI) ≥ 18 cm or single deepest pocket (SDP) ≥8 cm. |
| Assisted delivery | Forceps (9, 4.5 %); Ventouse (190, 95.5 %) |
| Chorioamnionitis | Chorioamnionitis has been defined as a clinical syndrome with any combination of fever, maternal or fetal tachycardia, uterine tenderness, foul-smelling amniotic fluid, or elevated white blood cell (WBC) count. |
| Induction of labor | Artificial membrane rupture (110,8.33 %); Mechanical method (84, 6.36 %);  Oxytocin (815,61.70%); Misoprostol (18,1.36%); Prostaglandin E2 (294,22.26%) |
| Placenta praevia | The placenta lies directly over the internal os. |
| Low lying placenta | The placental edge is less than 20 mm from the internal os on transabdominal or transvaginal scanning at more than 16 weeks of gestation |
| Uterus malformation | Bicornuate Uterus (2, 22.22 %); Septate uterus(7, 77.78 %); |

Abbreviations: PTB, preterm birth ; PE, Preeclampsia; GDM, gestational diabetes mellitus


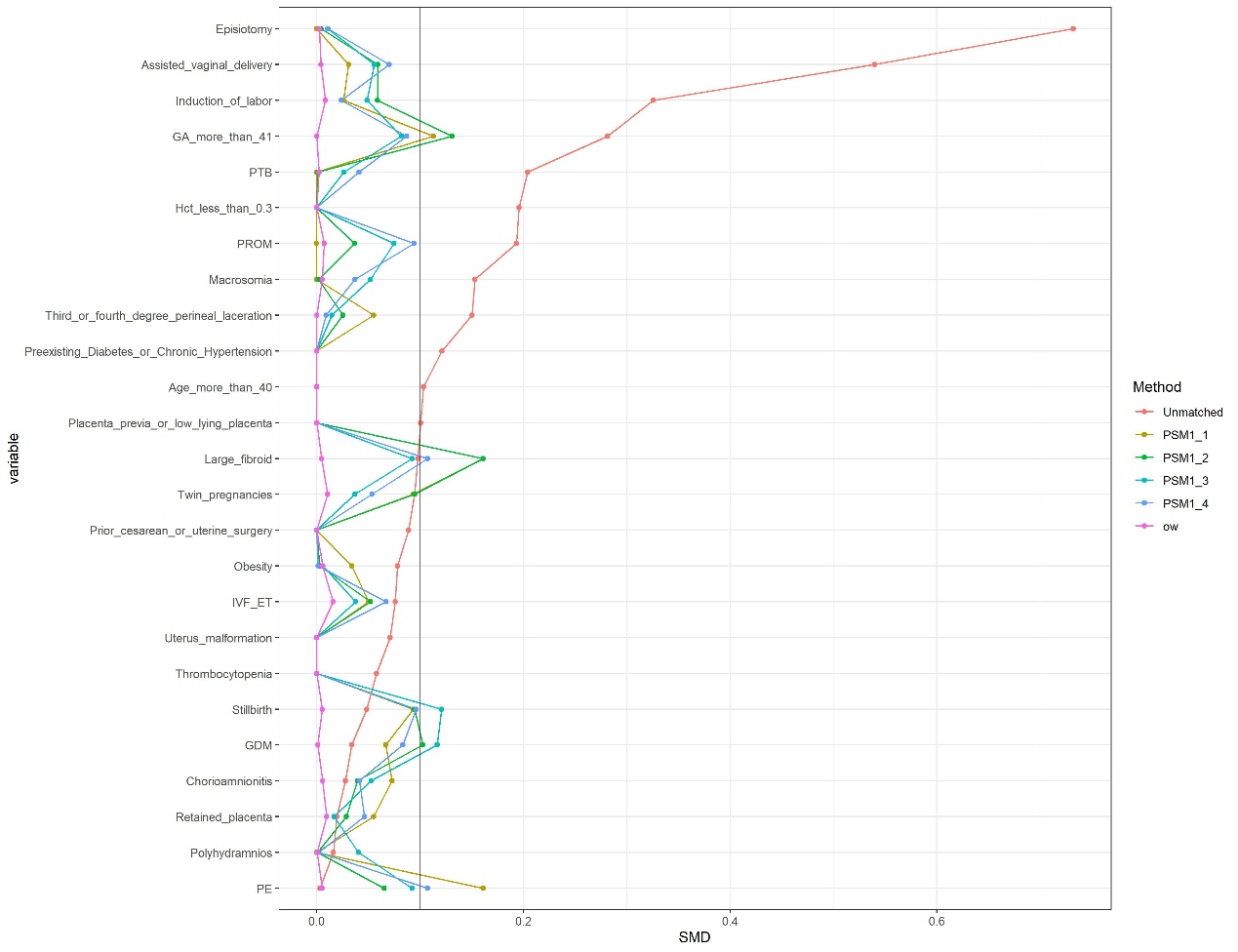


**Figure S1. Methods compared**

Abbreviations: PE, Preeclampsia; GDM, gestational diabetes mellitus; PTB, preterm birth; PROM, premature rupture of membrane.


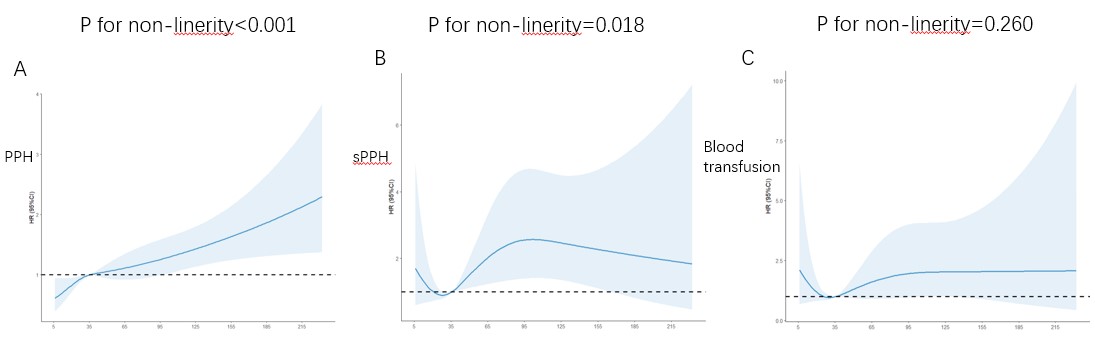


**Figure S2. The associations of second stage of labor with the outcome by restricted cubic spline regressions.** Solid lines are odds ratios, with light bands showing 95% confidence intervals derived from restricted cubic spline regressions with four knots at the 5th, 35th, 65th, and 95th percentiles of maternal age. Reference lines for no association are indicated by the dashed lines at an odds ratio of 1.0.

Abbreviations: PPH，postpartum hemorrhage; sPPH, severe postpartum hemorrhage
